# TOB1 modulates neutrophil phenotypes to influence gastric cancer progression and immunotherapy efficacy

**DOI:** 10.3389/fimmu.2024.1369087

**Published:** 2024-03-28

**Authors:** Jinfeng Zhang, Yunlong Li, Jing Chen, Tongtong Huang, Jing Lin, Yilin Pi, Huiting Hao, Dong Wang, Xiao Liang, Songbin Fu, Jingcui Yu

**Affiliations:** ^1^ Scientific Research Center, The Second Affiliated Hospital of Harbin Medical University, Harbin, Heilongjiang, China; ^2^ Department of General Surgery, The Second Affiliated Hospital of Harbin Medical University, Harbin, Heilongjiang, China; ^3^ Department of Gastroenterology, The Second Affiliated Hospital of Harbin Medical University, Harbin, Heilongjiang, China; ^4^ Department of Clinical Laboratory, Harbin Medical University Cancer Hospital, Harbin, Heilongjiang, China; ^5^ Key Laboratory of Preservation of Human Genetic Resources and Disease Control in China, Harbin Medical University, Ministry of Education, Harbin, China

**Keywords:** TOB1, gastric cancer, neutrophils apoptosis, immunotherapy, immune cells

## Abstract

**Introduction:**

The ErbB-2.1(TOB1) signaling transducer protein is a tumor-suppressive protein that actively suppresses the malignant phenotype of gastric cancer cells. Yet, TOB1 negatively regulates the activation and growth of different immune cells. Understanding the expression and role of TOB1 in the gastric cancer immune environment is crucial to maximize its potential in targeted immunotherapy.

**Methods:**

This study employed multiplex immunofluorescence analysis to precisely delineate and quantify the expression of TOB1 in immune cells within gastric cancer tissue microarrays. Univariate and multivariate Cox analyses were performed to assess the influence of clinical-pathological parameters, immune cells, *TOB1*, and double-positive cells on the prognosis of gastric cancer patients. Subsequent experiments included co-culture assays of si-*TOB1*-transfected neutrophils with AGS or HGC-27 cells, along with EdU, invasion, migration assays, and bioinformatics analyses, aimed at elucidating the mechanisms through which *TOB1* in neutrophils impacts the prognosis of gastric cancer patients.

**Results:**

We remarkably revealed that TOB1 exhibits varying expression levels in both the nucleus (nTOB1) and cytoplasm (cTOB1) of diverse immune cell populations, including CD8^+^ T cells, CD66b^+^ neutrophils, FOXP3^+^ Tregs, CD20^+^ B cells, CD4^+^ T cells, and CD68^+^ macrophages within gastric cancer and paracancerous tissues. Significantly, TOB1 was notably concentrated in CD66b+ neutrophils. Survival analysis showed that a higher density of cTOB1/nTOB1^+^CD66b^+^ neutrophils was linked to a better prognosis. Subsequent experiments revealed that, following stimulation with the supernatant of tumor tissue culture, the levels of TOB1 protein and mRNA in neutrophils decreased, accompanied by enhanced apoptosis. HL-60 cells were successfully induced to neutrophil-like cells by DMSO. Neutrophils-like cells with attenuated TOB1 gene expression by si-TOB1 demonstrated heightened apoptosis, consequently fostering a malignant phenotype in AGS and HCG-27 cells upon co-cultivation. The subsequent analysis of the datasets from TCGA and TIMER2 revealed that patients with high levels of TOB1 combined neutrophils showed better immunotherapy response.

**Discussion:**

This study significantly advances our comprehension of TOB1’s role within the immune microenvironment of gastric cancer, offering promising therapeutic targets for immunotherapy in this context.

## 1 Introduction

Gastric cancer is a malignancy characterized by a high incidence and mortality rate (1). Its pathogenesis is intricate, involving factors such as gene mutations, tumor suppressor gene inactivation, and inflammatory responses (2). In the treatment of gastric cancer, in addition to surgery, traditional radiotherapy, and chemotherapy, there is a growing focus on targeted immunotherapy. However, current immunotherapy drugs are not effective for all patients (3, 4), possibly because our understanding of the immune microenvironment in gastric cancer patients is not comprehensive enough, especially regarding the dual role of neutrophils in tumor growth (5). Therefore, it is essential to identify appropriate predictive and prognostic biomarkers for assessing the immune status of patients with gastric cancer.

TOB1 (transducer of ERBB2.1), a member of the TOB/BTG (B-cell translocation gene) anti-proliferative protein family (6), plays a crucial role in cell proliferation, apoptosis, and tumorigenesis. Recognized as an anti-proliferative and anti-tumorigenic factor, it impedes tumor cell proliferation, invasion, and migration, while fostering tumor cell apoptosis. This multifaceted action contributes to the restraint of both tumor initiation and progression. Relevant literature indicates that TOB1 has a tumor-suppressive effect in various cancer types. For instance, studies have found that TOB1 expression is downregulated in breast cancer (7, 8), lung cancer (9, 10), and gastric cancer (11), and its overexpression can inhibit tumor cell proliferation and induce apoptosis. Our research group previously discovered that TOB1 functions as a tumor suppressor in gastric cancer (12, 13). TOB1 is expressed in the cytoplasm and/or nucleus of gastric tumor cells, and its main anti-proliferative function is exerted in the nucleus (11). Additionally, Kundu et al. found that the anti-proliferative, anti-invasive, and anti-metastatic effects of TOB1 are achieved by binding with SMAD4 to inhibit the β-catenin signaling pathway (14). Phosphorylated TOB1 is an inactivated form (15), and our group revealed that phosphorylation of TOB1 at threonine 172 and serine 320 promotes aggressive phenotypes in gastric cancer (16). Moreover, high expression of phosphorylated TOB1 in the cell nucleus of patients with intestinal-type gastric cancer is associated with poor prognosis (11). In gastric cancer, the anti-tumor function of TOB1 is not only manifested through anti-proliferation but also involves inducing tumor cell autophagy. TOB1 promotes autophagy by inhibiting the AKT/mTOR signaling pathway (17). Alternatively, gastric tumor cells overexpressing TOB1 induce autophagy through the secretion of exosomes (18). Overall, TOB1 plays a crucial role in the anti-tumorigenic processes in gastric cancer.

During the immune response, the activation of T cells is a crucial step that enables them to recognize and respond to foreign antigens. However, to maintain the balance of the immune system, some T cells need to be in a non-responsive or dormant state, known as “anergic” or “quiescent” T cells (19). Under specific conditions, these cells are suppressed to avoid excessive immune responses and autoimmune reactions. TOB1, as a negative regulator, plays a significant role in these “anergic” or “quiescent” T cells (20, 21). It participates in signaling pathways that regulate the cell cycle, inhibiting the expression of cell cycle-dependent kinases and cell cycle-related proteins (cyclins), while reducing the transcription of IL-2. Additionally, it promotes the transcription of the cell cycle negative regulator p27^kip1^, resulting in cell cycle arrest at the G1 phase (22). As a result, cell proliferation is restricted, allowing these cells to remain in a non-responsive or quiescent state. Furthermore, TOB1 not only inhibits the proliferation of Th17 cells (23), but in inflammatory bowel disease, it also induces the expression of ID2 in CD4^+^ T cells via SMAD4/5 signaling, which suppresses the conversion of CD4^+^ T cells into Th1/Th17 cells, thereby inhibiting mucosal inflammation (24). This finding is consistent with the results observed in the experimental autoimmune encephalomyelitis Tob^-/-^ mouse model, where Tob^-/-^ mice showed an increase in CD4^+^ T, CD8^+^ T, Th1, and Th17 cell counts, but a decrease in FOXP3^+^ regulatory T cells (Tregs) (25).

In summary, TOB1 plays a role in adaptive immunity by inhibiting T cell proliferation and differentiation, thereby maintaining T cells in a quiescent state (22). In the context of innate immunity, TOB1 suppresses the production of IFN-β in macrophages, favoring viral replication (26). Therefore, we speculated that TOB1 acts as a negative regulator in immune responses. In gastric cancer, TOB1 inhibits tumor cell proliferation, invasion, and migration while promoting autophagy (11, 16–18), suggesting its potential as a therapeutic target for cancer treatment. However, understanding the role of TOB1 in the immune microenvironment of gastric cancer is crucial for targeted therapy. To clarify the impact of TOB1 in the gastric cancer immune microenvironment, this study used multiplex immunofluorescence (mIF) techniques to analyze TOB1 expression in gastric cancer tissues. Further exploration of the role of TOB1 in neutrophils in gastric cancer and its association with immunotherapy.

## 2 Results

### 2.1 Predicting *TOB1* expression in the immune cells in gastric cancer

**Figure 1:**
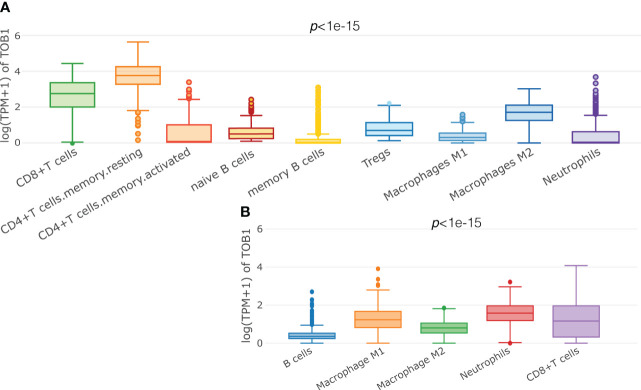
Predicting the expression of TOB1 in immune cells of gastric cancers. Boxplots were used to visualize the expression of log (TPM+1) TOB1 in each cell type selected, by CIBERSORT **(A)** and quanTIseq **(B)** algorithms.

A total of 415 tissue samples from patients with gastric cancer were used to analyze *TOB1* expression in immune cells, and the deconvolution algorithms CIBERSORT (**Figure 1A**) and quanTIseq (**Figure 1B**) from the GEPIA2021 website were used. ANOVA was used to reveal the differential expression of *TOB1* in various immune cells. The results showed varying levels of *TOB1* expression among different immune cells in gastric cancer, with a particularly noticeable pattern observed using the CIBERSORT algorithm. The highest trend median *TOB1* expression was observed in resting CD4^+^ T cells (median = 3.68), followed by CD8^+^ T cells (median = 2.84). Interestingly, using the quanTIseq algorithm, the highest trend median *TOB1* expression was observed in neutrophils (median = 1.577), followed by CD8^+^ T cells (median = 1.163). These results suggested that *TOB1* exhibited varying changes in expression at the mRNA level within the immune cells of gastric cancer.

### 2.2 Profiles of TOB1 expression and immune cell infiltration in gastric cancer

**Figure 2:**
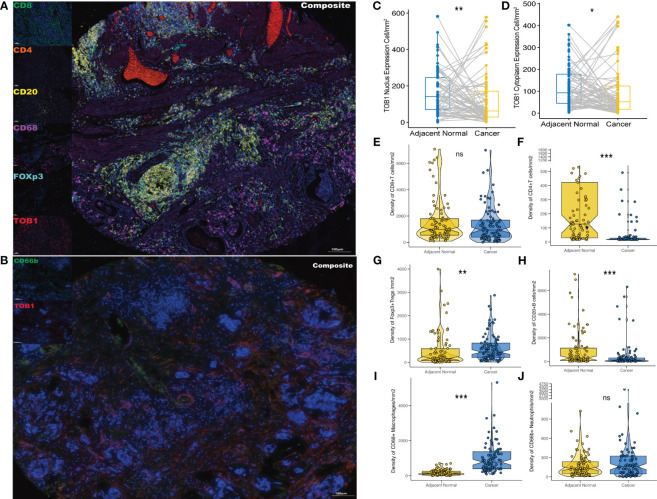
Expression profiles of TOB1 and immune biomarkers in gastric cancers. mIF images of representative gastric cancer sections analyzed for panel 1 **(A)** and panel 2 **(B)**. Paired boxplots were employed to show the density of nTOB1^+^ **(C)** and cTOB1^+^ **(D)** cells between cancer and adjacent paracancerous tissues by the paired Wilcoxon test analysis. Moreover, the box-violin plots depicted the densities of CD8^+^ T cells **(E)**, CD4^+^ T cells **(F)**, FOXP3^+^ Tregs **(G)**, CD20^+^ B cells **(H)**, CD68^+^ macrophages **(I)**, and CD66b^+^ neutrophils **(J)** between gastric cancer and the corresponding adjacent tissues. ^*^P < 0.05, ^**^P < 0.01, ^***^P < 0.001, ns, not significant.

Two TMA slides, each containing 90 pairs of gastric cancer and corresponding paracancerous tissues, were selected for mIF analysis of TOB1, CD8, CD4, FOXP3, CD20, CD68, and CD66b to investigate the profiles of TOB1 protein expression and immune cell infiltration in gastric cancer. In the mIF images, CD8, CD4, CD20, CD68, and CD66b were expressed on the cell membrane, whereas FOXP3 was expressed in the nucleus (**Figures 2A, B**). The corresponding IHC figure is shown in **Supplementary Figure S1**. However, TOB1 was expressed in both the cytoplasm and nucleus. Since the localization of TOB1 in the cells can affect its function, subsequent analyses were conducted separately for nuclear TOB1 (nTOB1) and cytoplasmic TOB1 (cTOB1). The results indicated that both nTOB1 and cTOB1 expression levels were lower in gastric cancer tissues than in paracancerous tissues (**Figures 2C, D**, P = 0.004 and P = 0.037, respectively). Regarding immune cells, the density of CD8^+^ T cells tended to be lower cancer tissues than in paracancerous tissues, with borderline significance (**Figure 2E**, P = 0.069). The density of CD4^+^ T cells and CD20^+^ B cells were lower in cancer tissues than in paracancerous tissues (**Figures 2F, H**, P < 0.001 and P < 0.001, respectively). The density of FOXP3^+^ Tregs and CD68^+^ macrophages were relatively higher in cancer tissues than in paracancerous tissues (**Figures 2G, I**, P = 0.002 and P < 0.001, respectively). The density of CD66b^+^ neutrophils was not significantly different between cancerous and paracancerous tissues (**Figure 2J**, P = 0.24). These findings offer an initial glimpse into the distribution of TOB1 expression and the immune cell infiltration in the context of gastric cancer.

### 2.3 TOB1 expression in the immune cells in gastric cancer

**Figure 3:**
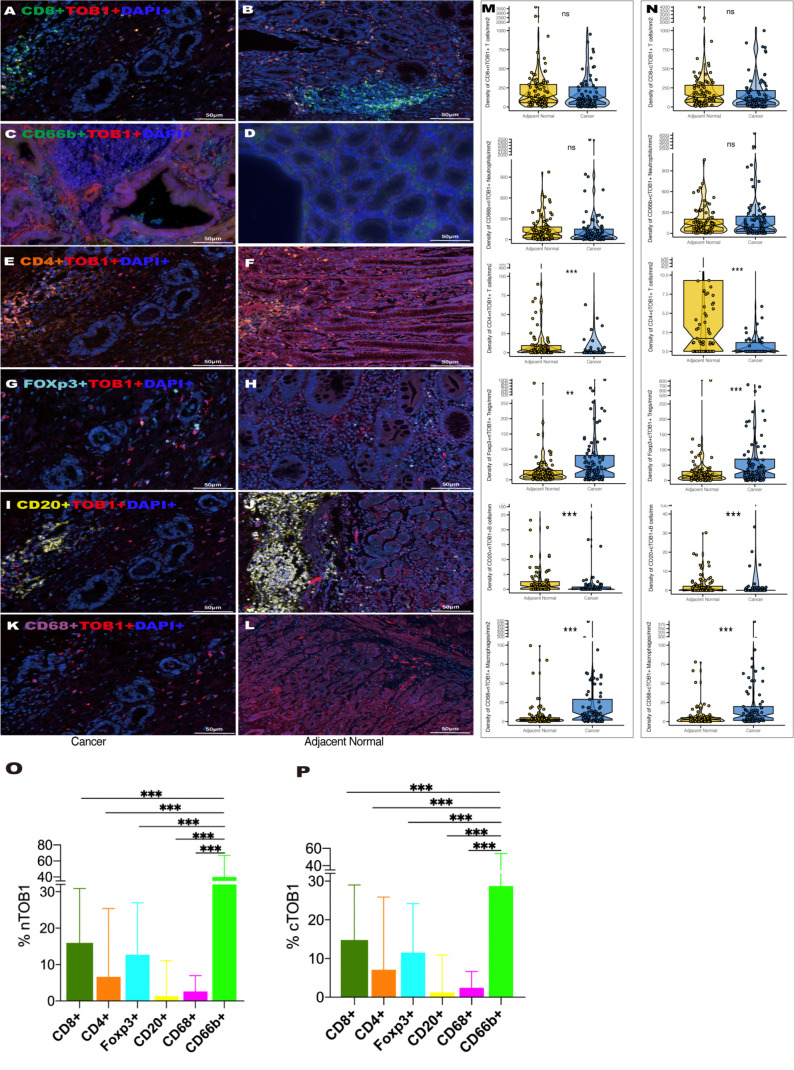
The expression of TOB1 in various immune cell types between gastric cancer and adjacent paracancerous tissues. TOB1 expression of CD8^+^ T cells **(A, B)**, CD66b^+^ neutrophils **(C, D)**, CD4^+^ T cells **(E, F)**, FOXP3^+^ Tregs **(G, H)**, CD20^+^ B cells **(I, J)**, and CD68^+^ macrophages **(K, L)**. The densities of double positive cells of each immune marker with nTOB1 **(M)** or cTOB1 **(N)** analyzing by pairwise Wilcoxon rank sum test. Bar graphs were employed to illustrate the expression proportions of nTOB1 **(O)** and cTOB1 **(P)** within various immune cells, with an ANOVA analysis conducted for assessment. ^**^P < 0.01, ^***^P < 0.001, ns, not significant.

The differences between nTOB1 and cTOB1 in cancerous and paracancerous tissues were analyzed by pairwise Wilcoxon rank sum test to investigate the expression and localization of TOB1 in the immune cells. positive images of TOB1 and each immune marker between cancerous and paracancerous tissues indicated that TOB1 was expressed in the nucleus and/or cytoplasm of the six immune cells (**Figures 3A–L**). A trend of lower densities of nTOB1^+^CD8^+^ T cells, cTOB1^+^CD8^+^ T cells, nTOB1^+^CD66b^+^ neutrophils, and cTOB1^+^CD66b^+^ neutrophils in cancerous tissues compared to paracancerous tissues (**Figures 3M, N**, P = 0.266, P = 0.189, P = 0.287, and P = 0.914, respectively). TOB1 was also detected in a small number of CD4^+^ T cells and CD20^+^ B cells. The densities of nTOB1^+^CD4^+^ T cells, cTOB1^+^CD4^+^ T cells, nTOB1^+^CD20^+^ B cells, and cTOB1^+^CD20^+^ B cells were significantly lower in cancer tissues than in paracancerous tissues (P < 0.001, P < 0.001, P < 0.001, and P < 0.001, respectively). In contrast, the densities of nTOB1^+^FOXP3^+^ Tregs, cTOB1^+^FOXP3^+^ Tregs, nTOB1^+^CD68^+^ macrophages, and cTOB1^+^CD68^+^ macrophages were higher in cancer tissues than in paracancerous tissues (P = 0.003, P < 0.001, P < 0.001, and P < 0.001, respectively). In gastric cancer tissues, comparative analysis was conducted using ANOVA to assess the proportion of positive cells for nTOB1 or cTOB1 across various immune cell types. Among these immune cell types, neutrophils exhibited the highest proportion of positive cells for both nTOB1 (**Figure 3O**, compared with each other group, P < 0.001) and cTOB1 (**Figure 3P**, compared with each other group, P < 0.001). Statistical analysis of pairwise differences between other each group was also conducted (**Supplementary Figures S2A, B**). These findings indicated that TOB1 was expressed across the six immune cell types, with the highest expression observed in CD66b^+^ neutrophils.

### 2.4 Correlation of TOB1 expression and immune cell infiltration in gastric cancer

**Figure 4:**
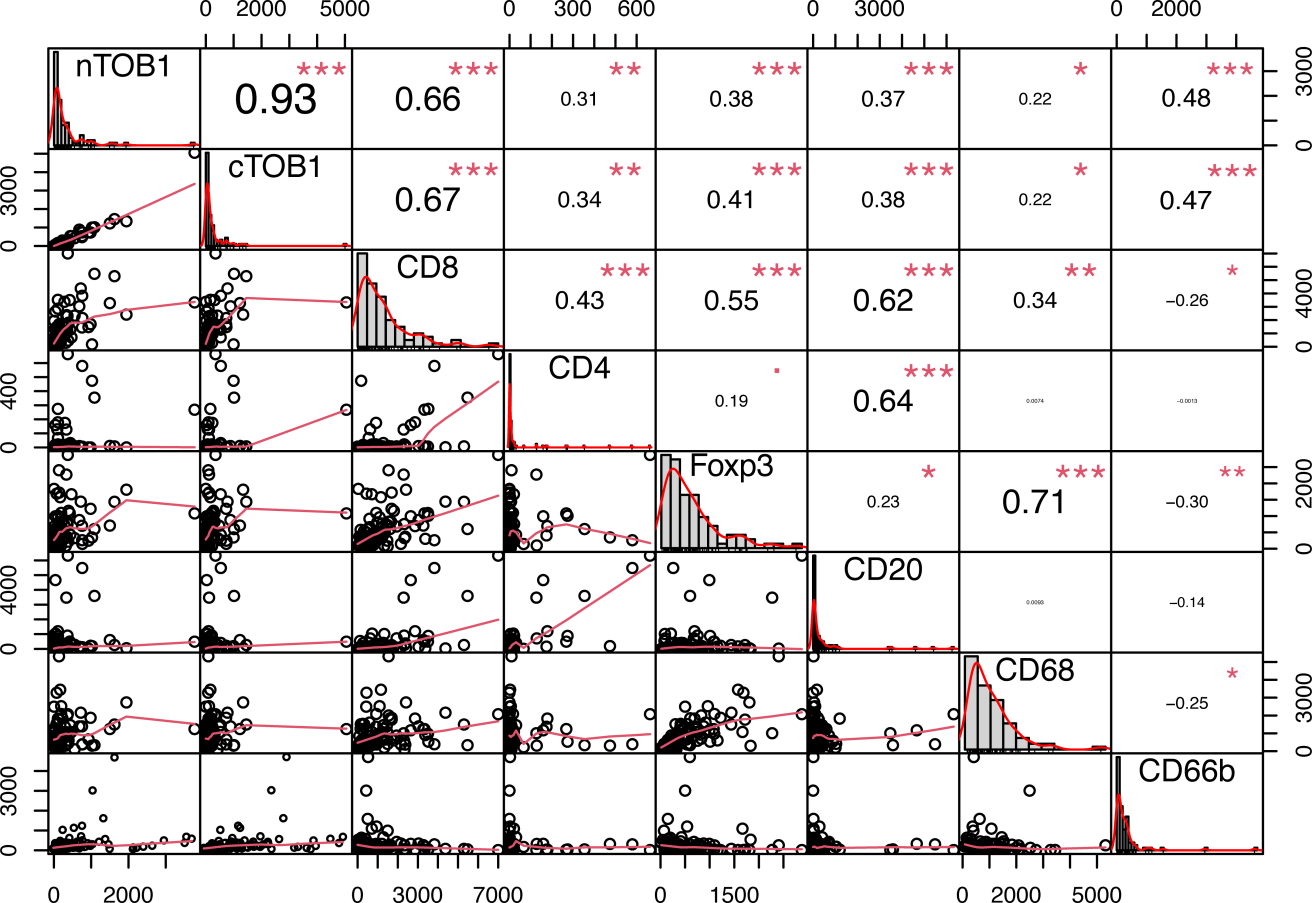
The correlation of TOB1 expression and immune cell infiltration in gastric cancer tissues. Correlation analysis between densities of nTOB1 or cTOB1 and each immune marker positive cells, with the method of Spearman. The lower left section of the figure displays scatter plots depicted the correlations between each pair of indicators. The upper right section provides correlation coefficient (R) values and significance levels. The diagonal section shows the distribution of each individual indicator in bar graphs. ^*^P < 0.05, ^**^P < 0.01, ^***^P < 0.001.

To explore the relationship between TOB1 and immune cells in gastric cancer, Spearman correlation analysis was performed for each marker, including nTOB1, cTOB1, CD8, CD4, FOXP3, CD20, CD68, and CD66b in cancerous and paracancerous tissues. There was a strong positive correlation between nTOB1 and cTOB1 expression in cancer tissues (R = 0.93, P < 0.001). Notably, in cancer tissues, the correlation between the densities of nTOB1 or cTOB1 and the density of CD8^+^ T cells was particularly significant (R = 0.66, P <0.001 and R = 0.67, P <0.001, respectively), followed by the densities of CD66b^+^ neutrophils (CD66b with nTOB1, R = 0.48, P <0.001; CD66b with cTOB1, R = 0.47, P <0.001), FOXP3^+^ Tregs (FOXP3 with nTOB1, R = 0.38, P <0.001; FOXP3 with cTOB1, R = 0.41, P <0.001), CD20^+^ B cells (CD20 with nTOB1, R = 0.37, P <0.001; CD20 with cTOB1, R = 0.38, P <0.001), CD4^+^ T cells (CD4 with nTOB1, R = 0.31, P = 0.003; CD4 with cTOB1, R = 0.34, P = 0.001), and CD68^+^ macrophages (CD68 with nTOB1, R = 0.22, P = 0.043; CD68 with cTOB1, R = 0.22, P = 0.046) in descending order (**Figure 4**). However, in paracancerous tissues, nTOB1 also showed a positive correlation with cTOB1 expression, the correlation coefficient R-value was 0.95 (**Supplementary Figure S3**). Moreover, TOB1 expression was only weakly positively correlated with the densities of CD8^+^ T cells and CD66b^+^ neutrophils and did not reach statistical significance in paracancerous tissues. Further, TOB1 showed a negative correlation with the densities of several other immune cells and only the densities of FOXP3^+^ Tregs (R = -0.18, P = 0.08, and R = -0.23, P = 0.03, respectively for nTOB1 and cTOB1), CD20^+^ B cells (R = -0.21, P = 0.045, and R = -0.17, P = 0.12, respectively for nTOB1 and cTOB1), and CD68^+^ macrophages (R = -0.29, P = 0.005, and R = -0.32, P = 0.003, respectively for nTOB1 and cTOB1) exhibited statistically significant differences. These findings revealed that while the expression level of TOB1 was higher in gastric paracancerous tissues than in gastric cancer tissues, higher TOB1 expression had a minimal correlation with immune cell infiltration. In contrast, in gastric cancer tissues, lower TOB1 levels were strongly associated with immune cell infiltration.

### 2.5 Clinical features of TOB1-positive immune cells and prognosis of patients with gastric cancer

**Figure 5:**
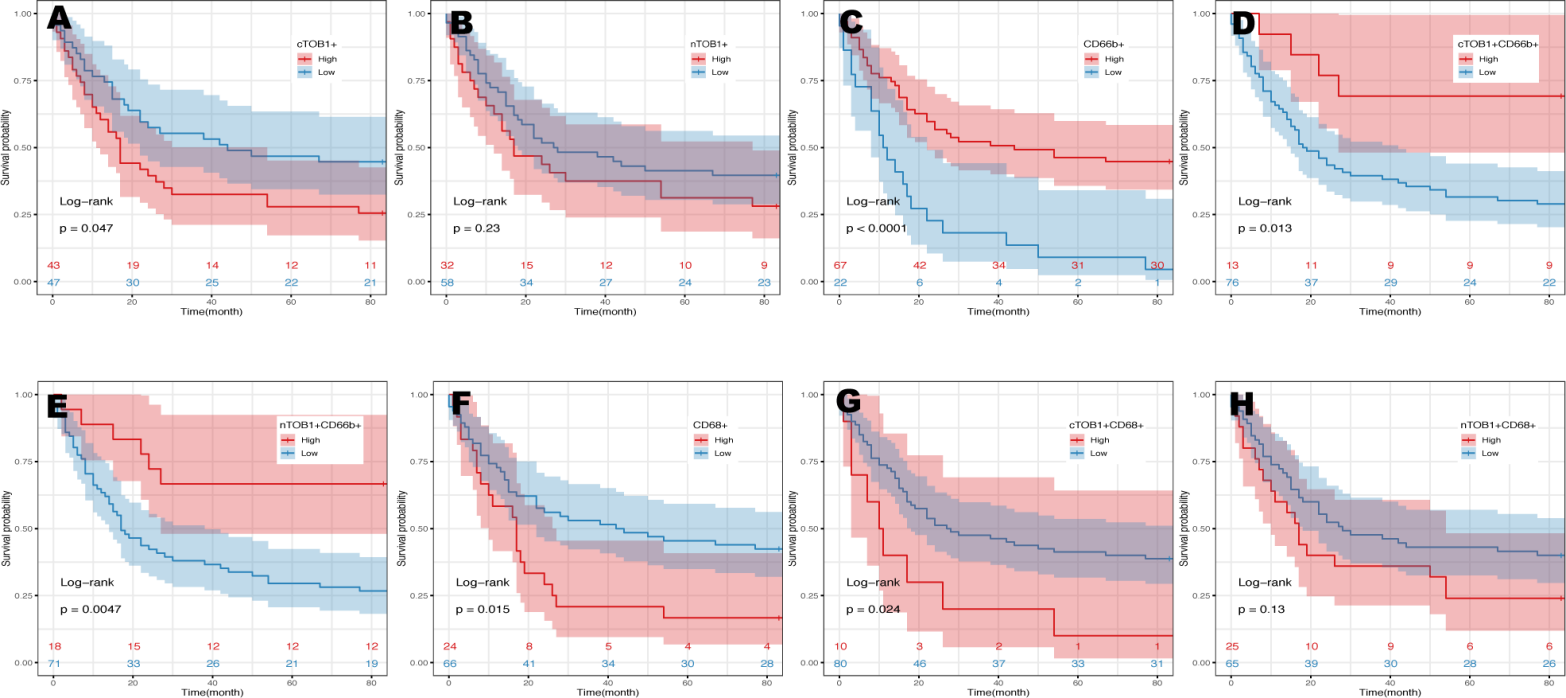
Kaplan-Meier analysis in gastric cancers. The prognosis of gastric cancer patients with the densities of cTOB1^+^ cells **(A)**, nTOB1^+^ cells **(B)**, CD66b^+^ neutrophils **(C)**, cTOB1^+^CD66b^+^ neutrophils **(D)**, nTOB1^+^CD66b^+^ neutrophils **(E)**, CD68^+^ macrophages **(F)**, cTOB1^+^CD68^+^ macrophages **(G)**, and nTOB1^+^CD68^+^ macrophages **(H)**. Highlighted lines represent subgroups with high (red line) and low (blue line) densities.

Survival analysis was performed using the R package survminer to calculate cutoff values to explore the potential impact of immune cell infiltration and TOB1 expression levels in immune cells on the prognosis of patients with gastric cancer. As shown in **Table 1** and **Supplementary Table S2**, the prognosis of patients with gastric cancer was influenced by clinical and pathological parameters, including tumor size, stage, lymph node metastasis, and TNM clinical stage (P = 0.003, P = 0.036, P = 0.000, and P = 0.000, respectively). Overall survival (OS) of patients with gastric cancer with low and high densities of cTOB1^+^, nTOB1^+^, CD66b^+^, cTOB1^+^CD66b^+^, nTOB1^+^CD66b^+^, CD68^+^, cTOB1^+^CD68^+^, and nTOB1^+^CD68^+^ cells was analyzed using Kaplan–Meier analysis. Of note, a higher density of cTOB1^+^ cells (**Figure 5A**, P = 0.047) was significantly associated with a shorter OS and was an independent prognostic factor (hazard ratio [HR] = 1.77, 95% confidence interval [CI]: 1.01–3.1, P = 0.045), whereas the impact of nTOB1^+^ cell density (**Figure 5B**, P = 0.23) on patient prognosis was not significant. For immune cells, patients with higher densities of CD66b^+^ neutrophils (**Figure 5C**, P < 0.001), nTOB1^+^CD66b^+^ neutrophils (**Figure 5D**, P = 0.005), and cTOB1^+^CD66b^+^ neutrophils (**Figure 5E**, P = 0.013) exhibited a better prognosis than patients with lower densities. Furthermore, multivariate cox regression analysis revealed that the densities of CD66b^+^ neutrophils (HR = 4.18, 95% CI:1.31–13.4, P = 0.016) and nTOB1^+^CD66b^+^ neutrophils (HR = 1.77, 95% CI:1.01–3.1, P = 0.045) were independent factors influencing patient prognosis. Patients with higher densities of CD68^+^ macrophages (**Figure 5F**, P = 0.015) and cTOB1^+^CD68^+^ macrophages (**Figure 5G**, P = 0.024) had poorer OS, whereas the density of nTOB1^+^CD68^+^ macrophages (**Figure 5H**, P = 0.13) did not significantly affect patient prognosis. Additionally, patients with a higher density of CD20^+^ B cells tended to have poorer survival rates. Further, densities of CD8^+^T cells, CD4^+^ T cells, FOXP3^+^ Tregs, and double-positive cell lines with nTOB1 or cTOB1 showed no significant impact on patient survival prognosis (**Supplementary Figure S4**). These results revealed the impact of cell infiltration of neutrophils and macrophages and TOB1 expression on the survival prognosis of patients with gastric cancer. It is important to note that not only did high-density nTOB1^+^CD66b^+^ neutrophils and cTOB1^+^CD66b^+^ neutrophils exhibit better survival prognosis, but the former also independently influenced patient prognosis.

**Table 1:** Univariate and multivariate cox regression analysis of gastric cancers.

Variables	Univariate Cox analysis				Multivariate analysis		
		HR	95% CI for HR	*P value*	HR	95% CI for HR	*P value*
Gender	n	1.18	0.69 - 2.03	0.551			
male	61						
female	29						
Age		0.62	0.36 - 1.05	0.073			
≥69	29						
<69	61						
Grade		1.31	0.91 - 1.91	0.151			
G1+G2	16						
G3	74						
Tumor size(cm)		0.45	0.27 - 0.77	0.003**	1.54	0.67 - 3.55	0.310
≥5.5	41						
<5.5	49						
Lauren’s classification		1.27	0.89 - 1.81	0.19			
Intestinal	37						
Diffuse	32						
Mixed	13						
Else	8						
T stage		4.52	1.1 - 18.54	0.036*	0.61	0.35 - 1.04	0.071
T1-T2	9						
T3-T4	81						
N stage		7.71	3.06 - 19.44	0.000***	1.53	0.34 - 6.93	0.583
N0	26						
≥N1	64						
M stage		5.22	0.69 - 39.39	0.109			
M0	89						
M1	1						
TNM stage		4.5	2.37 - 8.56	0.000***	4.18	1.31 - 13.4	0.016*
I-II	36						
III-IV	54						
nTOB1		0.72	0.43 - 1.23	0.231			
High	32						
Low	58						
cTOB1		0.59	0.35 - 1	0.049*	1.77	1.01 - 3.1	0.045*
High	43						
Low	47						
CD66b		2.91	1.68 - 5.02	0.000***	4.18	1.31 - 13.4	0.016*
High	67						
Low	22						
CD66b^+^cTOB1^+^		3.36	1.22 - 9.3	0.019*	1.54	0.67 - 3.55	0.310
High	13						
Low	76						
CD66b^+^nTOB1^+^		3.17	1.36 - 7.4	0.008**	1.77	1.01 - 3.1	0.045*
High	18						
Low	71						

^*^P < 0.05, ^**^P < 0.01, ^***^P < 0.001.

The P-values with statistical differences were highlighted in bold.

### 2.6 High TOB1 expression in peripheral blood neutrophils

**Figure 6:**
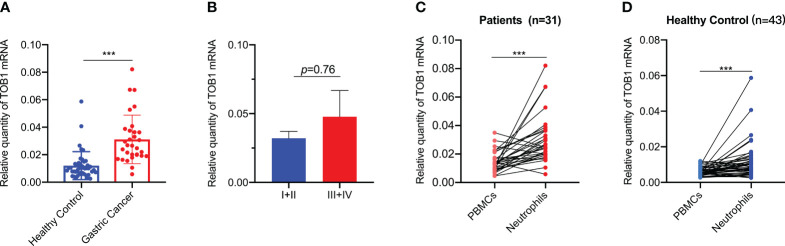
TOB1 expression in neutrophils and PBMCs of gastric cancer patients and healthy controls. **(A)** TOB1 mRNA of neutrophils in blood of gastric cancer patients and healthy control was examined by RT-qPCR. **(B)** The correlation between TOB1 mRNA expression level in neutrophils and TNM clinical stage (I+II vs III+IV) of gastric cancer patients was assessed using the Mann-Whitney test. The expression of TOB1 mRNA in both neutrophils and PBMCs from gastric cancer patients **(C)** and healthy controls **(D)** was assessed using the Wilcoxon matched-pairs signed rank test. ^***^P < 0.001.

TOB1 expression in peripheral blood neutrophils and peripheral blood mononuclear cells (PBMCs) from 31 patients with gastric cancer and 43 healthy individuals was analyzed. The neutrophils and PBMCs were isolated using density gradient centrifugation, and TOB1 mRNA levels were assessed by RT-qPCR. Interestingly, a significant upregulation of TOB1 mRNA in neutrophils was observed in gastric cancer patients when compared to the healthy control group (**Figure 6A**, P < 0.001). However, the TOB1 mRNA expression level was not significantly correlated with the TNM clinical staging of patients (**Figure 6B**, P = 0.76). In both patients with gastric cancer (**Figure 6C**, P < 0.001) and healthy controls (**Figure 6D**, P < 0.001), TOB1 mRNA expression in neutrophils was significantly higher than that in PBMCs. This finding was consistent with the results obtained from the tissue sample analysis. In previous results, we found a trend of lower TOB1 expression in neutrophils of gastric cancer tissue compared to adjacent non-cancerous tissue (**Figures 3M, N**). These observations prompted the hypothesis that the TOB1 expression levels may change upon neutrophils infiltration into tissues.

### 2.7 TOB1 promotes the antitumor activity of neutrophils and inhibits their apoptosis

**Figure 7:**
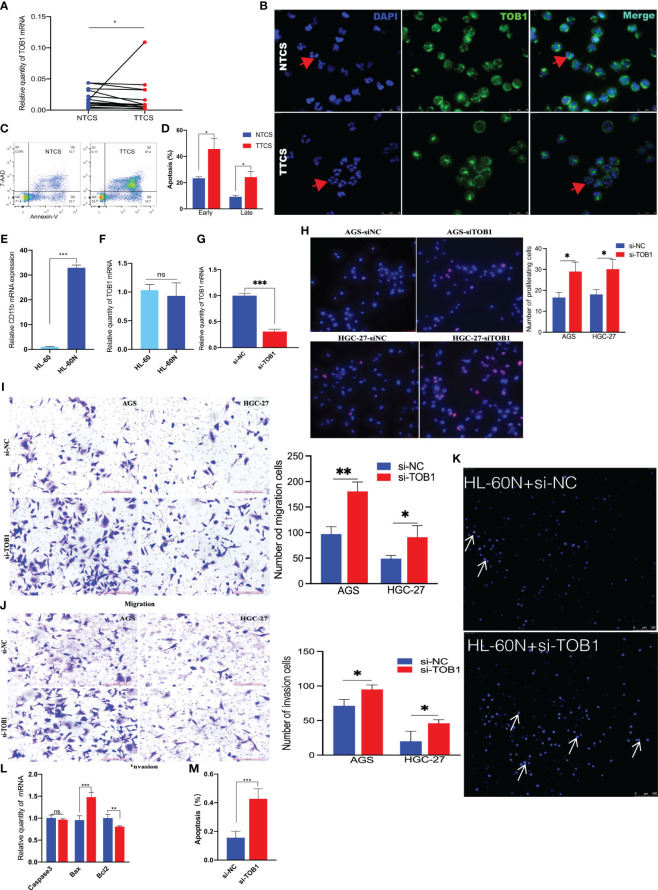
Evaluating the role of TOB1 in the antitumor function and apoptotic regulation of neutrophils. The TOB1 mRNA **(A)** and protein **(B)** expression levels in neutrophils following stimulation with NTCS or TTCS were assessed using RT-qPCR and immunofluorescence. **(C)** Neutrophils were cultured with NTCS or TTCS for 12 hours, and apoptosis was assessed using flow cytometry. **(D)** The data from three independent apoptosis experiments were collected and analyzed by paired t-tests. **(E)** After 4 days of DMSO induction, a notable upregulation of CD11b mRNA was observed in HL-60 cells. **(F)** No discernible alteration of TOB1 mRNA was demonstrated by comparative analysis before and after the induction of HL-60 cells by DMSO. **(G)** HL-60N cells were transfected with si-NC or si-TOB1 for 48h, mRNAs of TOB1 were detected by RT-qPCR. HL-60N transfected with si-TOB1 and si-NC were co-cultured with neutrophils and gastric cancer cell lines AGS and HGC-27 to evaluate their proliferation **(H)**, migration **(I)**, and invasion **(J)**. In figure H, pink represents proliferating cells, while blue represents cell nuclei. HL-60N cells were transfected with si-NC or si-TOB1 for 48h, cell apoptosis **(K)**, Caspase3, Bax, and Bcl2 **(L)** were detected by a apoptotic and necrosis assay kit and RT-qPCR, respectively. Additionally, quantitative analysis of apoptosis **(M)** was performed by randomly selecting three images from each of the three biological replicates per group (a total of nine images per group), counting the percentage of apoptotic cells, and conducting a t-test. Bright blue cells indicated by the arrows represented apoptotic cells, while red cells indicated necrotic cells. ^*^P< 0.05, ^**^P < 0.01, ^***^P< 0.001, ns, not significant.

Peripheral blood neutrophils obtained from healthy individuals were subjected to distinct stimuli using the tumor tissue culture supernatants (TTCS) and non-tumor tissue culture supernatants (NTCS). Subsequently RT-qPCR, immunofluorescence, and flow cytometry were used to assess the expressions of TOB1 mRNA and protein, as well as the apoptosis of stimulated neutrophils. Remarkably, a notable reduction in TOB1 mRNA (**Figure 7A**, P = 0.015) and TOB1 protein expressions (**Figure 7B**) was found, coupled with an increased incidence of cellular apoptosis (**Figure 7C**) in neutrophils from the TTCS groups, in contrast to the NTCS groups. Moreover, there was an increased number of hyper-segmented neutrophils (nucleus has ≥4 segments). Furthermore, significant differences were observed in the frequencies of early and late-stage apoptosis (**Figure 7D**, P = 0.048 and P = 0.039, respectively) in neutrophils between the TTCS and NTCS groups. Following tissue infiltration, a decrease in TOB1 expression in neutrophils was observed, resulting in the augmentation of neutrophil apoptosis. To investigate the role of TOB1 in the antitumor process of neutrophils, HL-60 cells were induced to differentiate into HL-60N using DMSO. Compared to HL-60 cells, a marked elevation in CD11b expression (**Figure 7E**, P< 0.001) was exhibited in HL-60N cells, with no substantial alteration in TOB1 mRNA (**Figure 7F**, P = 0.547). These results indicated that HL-60 cells were successfully induced into neutrophil-like cells. Following silencing of TOB1 in HL-60N cells through si-TOB1, with si-NC as the control group, the results revealed a significant reduction in TOB1 expression in the si-TOB1 group at the mRNA level compared to the si-NC control group (**Figure 7G**, P < 0.001). Upon co-culturing HL-60N cells transfected with si-TOB1 or si-NC, activated by TTCS stimulation, with AGS and HGC-27 cells for 48 hours, it was observed that the gastric cancer cells co-cultured with HL-60N cells from the si-TOB1 group exhibited enhanced proliferative (**Figure 7H**, P = 0.014 and P = 0.016), migratory (**Figure 7I**, P=0.004 and P=0.036), and invasive (**Figure 7J**, P =0.023 and P=0.045) capabilities. This suggests that TOB1 knockdown may contribute to the development of neutrophils into a phenotype that promotes gastric cancer. In tissue analysis, patients with neutrophils exhibiting high expression of TOB1 demonstrated better OS. To explore the underlying mechanisms, we examined apoptosis in HL-60N cells from the si-TOB1 and si-NC groups. Results revealed that, compared to the si-NC control group, TOB1 knockdown enhanced apoptosis in HL-60N cells (**Figure 7K**). This was associated with a decrease in the mRNA level of the anti-apoptotic factor Bcl2, an increase in the pro-apoptotic indicator Bax mRNA, and no significant change in Caspase3 levels in HL-60N cells (**Figure 7L**, P = 0.007, P < 0.001, and P = 0.893, respectively). Subsequent semi-quantitative analysis of the apoptosis results corroborated with the mRNA study, showing an increased percentage of apoptotic cells in the si-TOB1 group (**Figure 7M**, P < 0.001). These findings suggest that following tissue infiltration, there was a decrease in TOB1 expression in neutrophils, potentially resulting in a tumor-promoting phenotype accompanied by increased apoptosis.

### 2.8 Immunotherapy prediction of neutrophils with TOB1

**Figure 8:**
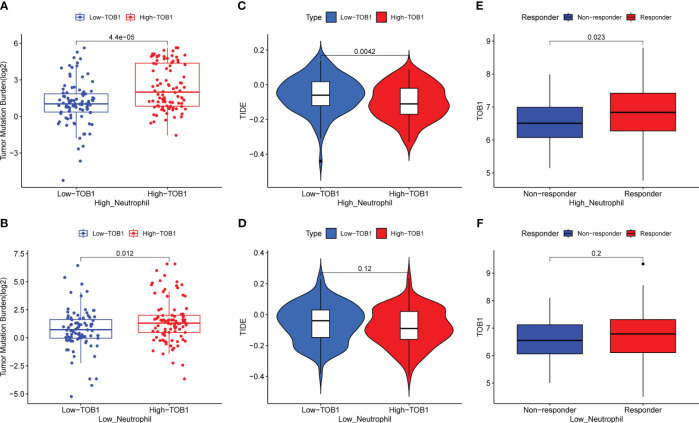
Evaluating the impact of TOB1 expression in neutrophils on immunotherapy in gastric cancer patients. **(A, B)** The boxplots of TMB differences between the high-TOB1 and low-TOB1 subgroups from the high-neutrophil and low-neutrophil groups, respectively. **(C, D)** The boxplots of TIDE differences between the high-TOB1 and low-TOB1 groups combined neutrophils. **(E, F)** The boxplots of TOB1 expression differences between immunotherapy response and non-response groups combined neutrophils in gastric cancer patients.

To further elucidate the mechanistic implications of TOB1 on the prognosis of gastric cancer patients, we compiled data from a cohort of 374 individuals with gastric cancer, sourced from the TCGA and TIMER2 databases. Employing neutrophils infiltration scores, the data were stratified into high and low neutrophils groups. Remarkably, regardless of whether it pertained to the high neutrophils group (**Figure 8A**, P < 0.001) or the low neutrophils group (**Figure 8B**, P = 0.012), the subgroup of high TOB1 exhibited an increased tumor mutation burden (TMB). Subsequent analysis revealed a noteworthy decrease in tumor immune dysfunction and exclusion (TIDE) within the high TOB1 subgroup when compared to the low TOB1 subgroup (**Figure 8C**, P = 0.004). In contrast, in the low neutrophils group, there was no statistically significant difference was observed between high and low TOB1 subgroups (**Figure 8D**, P = 0.12). Moreover, in the high neutrophils group, the high TOB1 subgroup exhibited significantly elevated immunotherapeutic responsiveness (**Figure 8E**, P = 0.023). And, in the low neutrophils group (**Figure 8F**, P = 0.2), although a similar trend was observed, no statistically significant difference was discerned. These results suggest that the expression level of TOB1 may influence the responsiveness of gastric cancer patients to immunotherapy and could potentially serve as a biomarker for immunotherapy responsiveness.

## 3 Discussion

Immune checkpoint inhibitors are among the most crucial immunotherapy agents and have shown substantial efficacy in select patients with gastric cancer (3, 4). However, owing to the inherent diversity of gastric cancer, responses to immunotherapy can vary significantly, which underscores the pressing need to identify biomarkers for predicting immune therapy responses and ascertain new therapeutic targets aimed at enhancing treatment outcomes. *TOB1* is a gene that exerts a negative regulatory effect on the progression of gastric cancer and holds a significant position with potential as a novel target in targeted gastric cancer therapy. However, within immune cells, TOB1 presents a distinct facet. TOB1 suppresses T cell proliferation, preserves T cell quiescence, dampens the differentiation of CD4^+^ T cells into Th1/Th2 subsets, and hampers macrophage activation (14, 23–27). Consequently, a comprehensive understanding of TOB1 expression and function within the gastric cancer immune microenvironment is warranted.

By combining insights from website predictions and previous research, we opted for CD8, CD4, FOXP3, CD20, CD68, and CD66b as markers to characterize cytotoxic T cells, Th cells, Tregs, B cells, macrophages, and neutrophils, respectively, to investigate the role of TOB1 in the context of the gastric cancer microenvironment. By leveraging the unique functions of TOB1 within both the nucleus and cytoplasm, this study distinguished between cytoplasmic and nuclear TOB1, enabling precise quantification of its expression within individual cells. In general, both nTOB1 and cTOB1 (**Figures 2C, D**) exhibit lower levels in gastric cancer tissues compared to adjacent paracancerous tissues, consistent with our previous findings (11, 13). When analyzing cancerous and paracancerous tissues separately, it became evident that the expression of nTOB1 took precedence, as illustrated in **Supplementary Figure S1H**.

Among the six immune cell types, TOB1 displayed the highest correlation with CD8^+^ T cells in gastric cancer tissues (**Figure 4**, R = 0.66 and R = 0.76). cTOB1/nTOB1 showed relatively diminished expression in CD4^+^ T cells and CD20^+^ B cells, both of which showed reduced infiltration in cancer tissues compared to that in paracancerous tissues. However, patients with a high density of CD20^+^ B cells have shorter survival, contradicting the results of previous studies (28, 29), which may be attributed to significant individual variations among patients, coupled with the limited sample size in this study. Our study revealed that both nTOB1^+^FOXP3^+^ Tregs and cTOB1^+^FOXP3^+^ Tregs exhibited increased levels in cancerous tissues compared to paracancerous tissues. Notably, a robust correlation between cTOB1/nTOB1 and FOXP3 expression was observed in the cancer tissues (R = 0.41 and R = 0.38). The observed trend in this correlation is consistent with prior research, where there was a reduction in both the quantity and proportion of Tregs in Tob1^-/-^ mice (25). Our results also revealed that the density of CD68^+^ macrophages in gastric cancer tissue was greater than that in the corresponding paracancerous tissues, and the expression of cTOB1/nTOB1 in CD68^+^ macrophages followed the same pattern. Moreover, this study further highlights that patients with gastric cancer with an elevated density of cTOB1^+^CD68^+^ macrophages experience unfavorable prognoses (P = 0.024, **Figure 5G**). Previous research has indicated that following viral infection, TOB1 dampens antiviral immune responses of macrophages by mediating IFN-β expression (26). Within the tumor microenvironment of gastric cancer, patients with high CD68^+^ macrophage expression have poorer prognosis (30–32). A possible reason for the similar effect of CD68^+^ macrophages and cTOB1^+^CD68^+^ macrophages on gastric cancer patient survival is that cTOB1 may not exert an anti-proliferative function.

Tzachanis et al. (22) found that TOB1 was expressed at higher levels in peripheral blood lymphocytes compared to immune tissues such as the spleen and lymph nodes. However, in this study, both nTOB1 and cTOB1 showed the highest proportions in neutrophils, among the various immune cells in gastric cancer tissues (**Figures 3O, P**). Moreover, in peripheral blood, the expression level of *TOB1* mRNA in neutrophils was higher than in corresponding mononuclear cells (**Figures 6C, D**). This prompted us to investigate whether TOB1 played a crucial role in augmenting their anti-tumor functions. The role of neutrophils in gastric cancer is intricate, as they can exert both anti-tumor immune effects (33), and under specific conditions, contribute to tumor development (34). In this study, patients with gastric cancer with an elevated density of CD66b^+^ neutrophils exhibited better prognosis, aligning with prior research findings (33), albeit contradicting the conclusions of Lid et al. (35) Moreover, the increased expression of cTOB1/nTOB1^+^CD66b^+^ neutrophils has been linked to a favorable patient prognosis. Furthermore, through multivariate analysis, it has been established that nTOB1^+^CD66b^+^ neutrophils serve as an independent prognostic indicator, with an HR of 1.77 and a 95% CI of 1.01–3.1 (*P* = 0.045, **Table 1**). To gain further insight into the role of TOB1 in neutrophils within the immune microenvironment of gastric cancer, we conducted a comparative analysis of *TOB1* mRNA in peripheral blood neutrophils from patients with gastric cancer and healthy controls and found a significant elevation in *TOB1* mRNA levels in the peripheral blood neutrophils of patients (**Figure 6A**). Considering the alterations that neutrophils undergo upon entering the tumor microenvironment, we simulated the microenvironments of both gastric cancer and paracancerous tissues using TTCS and NTCS. We noted a decrease in both TOB1 protein and mRNA expression levels, along with increased neutrophils apoptosis following TTCS stimulation compared to the NTCS group (**Figures 7A–D**). Wang et al.’s study found that TTCS-activated neutrophils exhibit an immunosuppressive phenotype, impeding gastric cancer progression via the GM-CSF-PD-L1 pathway, along with reduced neutrophils apoptosis (36). To investigate the influence of TOB1 on phenotypes and apoptosis of neutrophils while excluding the impact of TTCS, HL-60N cells were transfected with si- *TOB1* and si-NC, followed by stimulation with TTCS, and subsequently co-cultured with AGS and HGC-27 cells. Compared to the si-NC control group, co-culturing with the si-*TOB1* group results in enhanced malignant phenotypes of tumor cells, coupled with increases apoptosis of neutrophil-like cells (**Figures 7H–M**). In tissue analyses, we observed that patients with a high expression of cTOB1/nTOB1^+^CD66b^+^ neutrophils had a better prognosis (**Figures 5D, E**). Therefore, we have reason to speculate that TOB1 enhanced patient prognosis by enhancing polarizing of neutrophils toward to anti-tumor phenotype and inhibiting their apoptosis. In other words, TOB1 extends the lifespan of neutrophils, leading to their accumulation, potentially facilitating the interaction between neutrophils and tumor cells. This interaction may result in direct tumor cell killing through antibody-dependent cellular cytotoxicity (37).TOB1 may regulate the TGF-β pathway in activated T cells by inhibiting twisted gastrulation (38), and there was substantial evidence to confirm that TGF-β induction led neutrophils towards a pro-tumor phenotype within the tumor microenvironment (39, 40). We speculate that *TOB1*, as an immune regulatory gene, may influence the reactivity of immunotherapeutic responses. In our study, gastric cancer patients with the high level TOB1 in neutrophils group may be more likely to benefit from immunotherapy (**Figures 8A, C, E**). Our experimental results align with previous studies, confirming that the elevated TMB is typically associated with increased tumor immunogenicity (41), while the decreased Tumor TIDE score may reflect a reduction in tumor immune evasion (42). As a pivotal breakthrough in cancer treatment, immunotherapy holds significant promise. The expression levels of TOB1 in neutrophils can serve as predictive indicators for patient response to immunotherapy, offering tangible benefits for patient treatment (43). In this study, the favorable prognosis of patients with a high density of TOB1^+^CD66b^+^ neutrophils might be due to alterations in TOB1 expression levels upon neutrophil infiltration into the tissue, leading to changes in neutrophils apoptosis and subsequently influencing tumor progression, even by modulating immunotherapy to enhance the prognosis of gastric cancer patients. However, further investigation is needed to confirm this hypothesis.

## 4 Conclusions

This study innovatively analyzed the expression of nTOB1 and cTOB1 in immune cells within gastric cancer tissues using mIF. High expression of TOB1 in CD66b^+^ neutrophils was identified. Additionally, high densities of nTOB1^+^CD66b^+^ neutrophils and cTOB1^+^CD66b^+^ neutrophils were associated with a favorable prognosis in patients with gastric cancer, and the former serves as an independent prognostic indicator. The findings indicate that TOB1 potentially extends the survival of gastric cancer patients by fostering the anti-tumor polarization of neutrophils, restraining their apoptosis, and augmenting patients’ responsiveness to immunotherapy. For further validation of the mechanism underlying TOB1 in neutrophils and its predictive responsiveness to immunotherapy, *in vivo* experimentation utilizing mouse models is imperative.

## 5 Materials and methods

### 5.1 Tissues and blood and TMAs

All patients with gastric cancer and healthy individuals were recruited from the Affiliated Cancer Hospital of Harbin Medical University and the Affiliated Second Hospital of Harbin Medical University between January 2019 and December 2020. Tissue samples were collected, including 15 pairs of cancer and adjacent normal tissues (at least 5 cm from the cancer site), from patients with gastric cancer who underwent surgical resection. Peripheral blood samples were collected from 31 patients with gastric cancer and 43 healthy individuals. Healthy volunteers with infectious diseases, autoimmune diseases, or multiple primary cancers were excluded from the study. The tissue microarrays (TMAs, HStm-A180Sur-11, ethics no. XT17-035) consisting of 90 gastric cancer tissues and their corresponding adjacent noncancerous tissues were purchased from Shanghai Outdo Biotech Co. Ltd. The core diameter on each TMA was 1.5 mm, ensuring a greater quantity of representative tissues on the TMAs. The clinical stages of gastric cancer were determined based on the tumor, node, metastases (TNM) classification system of the International Union Against Cancer (8th edition). Among the 90 samples, 37 cases were classified as intestinal-type, 32 as diffuse type, 13 as mixed type, and 8 were undefined according to the Laurén classification. Additionally, 30-point gastric cancer tissue TMAs (HStm-Ade030PG-01, kindly gifted by Shanghai Outdo Biotech Co., Ltd.) were used for pre-tests of mIF. None of the patients received pre-operative chemotherapy or radiotherapy.

### 5.2 Predicting TOB1 expression in the immune cells of gastric cancer

To assess the TOB1 expression levels in immune cells, such as CD8^+^ T cells, CD4^+^ T cells, Tregs, B cells, M1 and M2 macrophages, and neutrophils, within the gastric cancer microenvironment, we employed two algorithms, CIBERSORT and quanTIseq, which were performed on a cohort of 415 patients with gastric cancer from The Cancer Genome Atlas database using the GEPIA2021 (44) web platform (http://gepia2021.cancer-pku.cn/sub-expression.html).

### 5.3 Multiplex immunohistochemistry

Two panels were used to investigate TOB1 expression and the subsets of immune cells associated with gastric cancer. Panel 1 consisted of markers for TOB1, CD8, CD4, FOXP3, CD20, and CD68, while Panel 2 included markers for TOB1 and CD66b. Before conducting the formal mIF experiments, 30-point TMAs were used for regular immunohistochemistry (IHC) and uniplex immunofluorescence to optimize the staining conditions, following previously published instructions. IHC was performed to verify the efficacy of antibodies and determine initial dilution ratios for each marker: CD8 (ZA-0508; Zsbio, China, 1:200), CD4 (ab133616; Abcam, USA, 1:200), FOXP3 (ab4728; Abcam, 1:100), CD20 (ab9475; Abcam, 1:200), CD68 (ab955; Abcam, 1:200), CD66b (555723; BD Biosciences, USA, 1:1000). For the TMA slides, uniplex and multiplex immunofluorescence staining were performed using the Opal7-Color Fluorescent IHC Kit (NEL811001KT; PerkinElmer, Massachusetts, USA) incorporating fluorophores, including DAPI. The Opal protocol was used, with specific adjustments for enhanced precision and efficacy. The optimized tagging sequence for Panel 1was as follows: anti-TOB1 (Opal 520), anti-CD4 (Opal 620), anti-CD8 (Opal 540), anti-CD20 (Opal 570), anti-FOXP3 (Opal 650), and anti-CD68 (Opal 690). The staining sequences for Panel 2 were anti-CD66b (Opal 520) and anti-TOB1 (Opal 620). Finally, the slides were counterstained with DAPI for 5 min and mounted using the VECTASHIELD Hard Set (H-1400; Vector Labs, Burlingame, CA, USA). Stained slides were scanned, and images were analyzed using the Vectra Polaris multispectral slide imaging system and inForm tissue finder image analysis software (inForm 2.3.0; PerkinElmer, Massachusetts, USA). The density of the positively stained cells was quantified as the number of cells per mm^2^.

### 5.4 Preparation of TTCS and NTCS

TTCS and NTCS were prepared following the protocol described by Li et al (36, 45). Subsequently, the supernatant was centrifuged and filtered through a 0.22 μm membrane before being harvested and stored at -80°C.

### 5.5 Neutrophils isolation and culture with TTCS or NTCS

Following collection, 5–10 ml of EDTA-anticoagulated peripheral blood samples were promptly transferred to the laboratory for analysis within 2 h. In a 15 ml centrifuge tube, the layers were carefully arranged as follows: 2.5 ml of Histopaque 1.119, 2.5 ml of Histopaque 1.077 (Sigma-Aldrich, St. Louis, MO, USA), and 5 ml of mixed whole blood. The tube was then centrifuged at 700 g for 30 min at room temperature, resulting in the formation of a primarily neutrophil layer positioned between the Histopaque 1077 and 1119 layers. To obtain purified neutrophils and PBMCs, any residual erythrocytes were removed using Red Cell Lysis Buffer (TIANGEN RT122-02). The purity of the isolated neutrophils was evaluated through Giemsa staining, wherein neutrophils were counted in 10 high-power fields under a microscope, and the neutrophils ratio was calculated as a proportion of all cells. Furthermore, the percentage of CD66b^+^ neutrophils was assessed using flow cytometry to further evaluate the purity of neutrophils. Neutrophils with a purity of over 95% were used for subsequent experiments (data not shown). To generate conditioned neutrophils, peripheral blood neutrophils from healthy individuals were cultured with 50% TTCS or NTCS at a density of 1 × 10^6^ cells/mL for 12 h, allowing for the development of conditioned neutrophils.

### 5.6 Immunofluorescence

Immunofluorescence was used to compare the changes in TOB1 protein expression in neutrophils following stimulation with TTCS or NTCS. Conditioned neutrophils were harvested and centrifuged, and the cells were washed three times with PBS. Subsequently, the cells were fixed with 4% paraformaldehyde at room temperature for 20 min and a cell suspension of 5 × 10^5^ cells/ml prepared. Next, 100 μl of the cell suspension was added to alcohol pre-treated slides, and the cell smears centrifuged at 3000 rpm for 5 min in a cell smearing centrifuge. The subsequent steps were carried out as for regular immunofluorescence staining, including blocking, incubation with the primary antibody (TOB1, diluted 1:200), incubation with Alexa Fluor TM488 goat anti-mouse IgG antibody (diluted 1:300), and finally sealing the smear with DAPI. The results were then observed under a fluorescence microscope.

### 5.7 Induction and transfection of neutrophil-like cells

The human promyelocytic leukemia cell line (HL-60) was purchased from iCell Bioscience Inc. (Shanghai, China). HL-60 cells were induced to differentiate into neutrophil-like cells (HL-60N) by culturing them in RPMI-1640 medium supplemented with 10% FBS and 1.25% DMSO (Sigma-Aldrich) for 4 days (46). For the transfection experiment, HL-60N cells were seeded at a density of 5 × 10^5^ cells/well in plates and incubated for 48 h in the presence of 100 nM TOB1 small interfering RNA (si- *TOB1*) or negative control siRNA (si-NC) obtained from General Biol (Chuzhou, China) and the Lipofectamine 2000 transfection reagent (Invitrogen, Carlsbad, CA, USA). All cells were maintained in a humidified incubator at 37°C with 5% CO_2_. The sequences of si- *TOB1* and si-NC are provided in **Table S1**.

### 5.8 Co-culture of neutrophil-like cells and tumor cells

Logarithmically growing HGC-27 or AGS cells were seeded in the lower chamber, followed by the addition of previously stimulated HL-60N+si- *TOB1* or HL-60N+si-NC cells by TTCS into the upper chamber for incubation for 48 hours.

### 5.9 Apoptosis assessment

TTCS/NTCS-conditioned neutrophils were suspended in binding buffer to create a cell suspension. Annexin V-FITC and/or 7-amino actinomycin D (7-AAD) were added to the suspension and gently mixed. The mixture was then incubated for 15 min at room temperature and protected from light. Within 1 h, a specific system of binding buffer was added to each tube before performing flow cytometry. The apoptosis of HL-60N cells transfected with si-*TOB1* or si-NC was evaluated using the Apoptosis and Necrosis Assay Kit (Beyotime, Nanjing, China) following the manufacturer’s instructions.

### 5.10 Quantitative real-time PCR

Total RNA was isolated from neutrophils and PBMCs of both patients with gastric cancer and healthy individuals, as well as from conditioned neutrophils, HL-60, HL-60N, and transfected HL-60N cells using the High Pure RNA Isolation Kit (Cat. No. 11828665001; Roche, Mannheim, Germany) following the manufacturer’s protocol. Subsequently, 3 μl of extracted RNA was reverse transcribed into cDNA using the Transcriptor First Strand cDNA Synthesis Kit (Cat. No. 04897030001; Roche). For mRNA quantification, real-time PCR was conducted using the Light Cycler 480 SYBR Green Kit (Roche) according to the manufacturer’s guidelines. β-actin served as the endogenous reference gene. Data were assessed using the comparative 2^-ΔCt^ or 2^-ΔΔCt^ method. The primer sequences used are provided in **Supplementary Table S1**.

### 5.11 Transwell and EdU proliferation assays

AGS or HGC-27 cells were seeded in the upper chamber with 200 μl of serum-free RPMI1640 medium, while 600 μl of 10% fetal bovine serum was added into the lower chamber. After incubating for 24 hours, non-migrated cells were gently wiped off from the Matrigel membrane, followed by fixation with 4% paraformaldehyde and staining with crystal violet. Observations and photography were conducted under a microscope. The experimental procedure for invasion was akin to that of migration experiments, except that the migration chamber was substituted with an invasion chamber coated with Matrigel mix. Proliferation of AGS and HGC-27 cells were assessed according to the instructions of the EdU cell proliferation assay kit (C0085S, Beyotime, Nanjing, China).

### 5.12 Predict the immunotherapy response of gastric cancer patients

The TPM formatted RNA sequencing data of TMB, and clinical information for gastric cancer were extracted from the TCGA database (https://www.cancer.gov/ccg/research/genome-sequencing/tcga), while the immune cell infiltration data were obtained from the TIMER2 database (http://timer.cistrome.org/). The CIBERSORT method (47) was employed to calculate the neutrophils infiltration scores for the gastric cancer tissues of each patient. Subsequently, patients were categorized into two groups: high neutrophils infiltration (n=187) and low neutrophils infiltration (n=187), with the classification based on the median neutrophils scores. The subgroup with high neutrophils infiltration was subsequently stratified into two subgroups: high *TOB1* expression (n=93) and low *TOB1* expression (n=94) based on the median *TOB1* expression levels. Similarly, the subgroup with low neutrophil infiltration was divided into high *TOB1* expression (n=93) and low *TOB1* expression (n=94) groups. The TIDE scores for patients with gastric cancer were derived using the TIDE online algorithm, accessible at http://tide.dfci.harvard.edu/.

### 5.13 Statistical analyses

In the first TMA slide, one paracancerous spot was excluded from the analysis due to being out of the scanning range, leaving 89 pairs of tissues available for paired analysis. Additionally, 90 cases were used for survival analysis. In the second TMA slide, there were two non-paired paracancerous spots and one cancerous spot that were out of the scanning range. This resulted in 87 pairs of tissues available for paired analysis, and 89 cases were used for survival analysis specifically focusing on CD66b. R package (version 4.1.2) was used to analyze the correlations between TOB1 and immune cells in gastric cancer and paracancer tissues. For log-rank and Cox regression analyses, the cutoff values for various indicators were calculated using the “surv_cutpoint” function from the R package survminer (version 0.4.9). The Spearman rank correlation test was conducted using the R package Performance Analytics (version 2.0.4). During this analysis, correlation coefficients and their corresponding *p*-values were calculated, correlations were visualized using dot-line charts, and the criteria for determining the strength of the correlation were as described previously (48).

## Data availability statement

The original contributions presented in the study are included in the article/**Supplementary Material**. Further inquiries can be directed to the corresponding author.

## Ethics statement

The studies involving humans were approved by The Ethics Committee of the Second Hospital of Harbin Medical University. The studies were conducted in accordance with the local legislation and institutional requirements. The participants provided their written informed consent to participate in this study.

## Author contributions

JZ: Writing – review & editing, Writing – original draft, Visualization, Validation, Supervision, Software, Resources, Methodology, Investigation, Data curation. YL: Writing – review & editing, Resources, Data curation. JC: Writing – review & editing, Resources. TH: Writing – review & editing, Validation, Data curation. JL: Writing – review & editing, Resources, Data curation. YP: Writing – review & editing, Resources, Data curation. HH: Writing – review & editing, Resources, Data curation. DW: Writing – review & editing, Supervision. XL: Writing – review & editing, Supervision. SF: Writing – review & editing, Supervision, Project administration. JY: Writing – review & editing, Supervision, Project administration, Investigation, Funding acquisition.
