# Supplementary material for: TOB1 modulates neutrophil phenotypes to influence gastric cancer progression and immunotherapy efficacy
Source: Front Immunol. 2024 Mar 28;15:1369087. doi: 10.3389/fimmu.2024.1369087 (PMC11010640; doi:10.3389/fimmu.2024.1369087)
Supplement: Supplementary file 1 [file DataSheet_1.docx]

**Supplement tables**

**Table S1.** The sequences of primers.

| Primers | Sequences |
| --- | --- |
| TOB1-F | GGTGAAAAGGGACCAGTGAA |
| TOB1-R | TGGAGAGCTGGACACTGATG |
| caspase 3-F | CATGGAAGCGAATCAATGGACT |
| caspase 3-R | CTGTACCAGACCGAGATGTCA |
| BAX-F | CCCGAGAGGTCTTTTTCCGAG |
| BAX-R | CCAGCCCATGATGGTTCTGAT |
| BCL2-F | GGTGGGGTCATGTGTGTGG |
| BCL2-R | CGGTTCAGGTACTCAGTCATCC |
| β-actin-F | ATCATGTTTGAGACCTTCAA |
| β-actin-R | CATCTCTTGCTCGAAGTCCA |
| sense si-TOB1 | GCUGUAAGCCCUACCUUCATT |
| antisense si-TOB1 | UGAAGGUAGGGCUUACAGCTT |
| sense si-NC | UUCUCCGAACGUGUCACGUTT |
| antisense si-NC | ACGUGACACGUUCGGAGAATT |

**Table S2.** Univariate and multivariate cox regression analysis of gastric cancers.

| Variables | **Univariate Cox analysis** | | | | | **Multivariate analysis** | | | |
| --- | --- | --- | --- | --- | --- | --- | --- | --- | --- |
|  |  |  | HR | p | CI |  | HR | CI | P |
| CD8^+^ |  |  | 0.58 | 0.055 | 0.34 - 1.01 |  |  |  |  |
|  | High |  |  |  |  |  |  |  |  |
|  | Low |  |  |  |  |  |  |  |  |
| nTOB1^+^CD8^+^ |  |  | 0.66 | 0.143 | 0.38 - 1.15 |  |  |  |  |
|  | High | 55 |  |  |  |  |  |  |  |
|  | Low | 35 |  |  |  |  |  |  |  |
|  |  |  |  |  |  |  |  |  |  |
| cTOB1^+^CD8^+^ |  |  | 0.62 | 0.077 | 0.36 - 1.05 |  |  |  |  |
|  | High | 50 |  |  |  |  |  |  |  |
|  | Low | 40 |  |  |  |  |  |  |  |
| CD4^+^ |  |  | 0.61 | 0.071 | 0.35 - 1.04 |  |  |  |  |
|  | High | 52 |  |  |  |  |  |  |  |
|  | Low | 38 |  |  |  |  |  |  |  |
|  |  |  |  |  |  |  |  |  |  |
| nTOB1^+^CD4^+^ |  |  | 1.76 | 0.276 | 0.64 - 4.86 |  |  |  |  |
|  | High | 9 |  |  |  |  |  |  |  |
|  | Low | 81 |  |  |  |  |  |  |  |
| cTOB1^+^CD4^+^ |  |  | 1.43 | 0.446 | 0.57 - 3.58 |  |  |  |  |
| Variables | **Univariate Cox analysis** | | | | | **Multivariate analysis** | | | |
|  |  |  | HR | p | CI |  | HR | CI | P |
|  | High | 10 |  |  |  |  |  |  |  |
|  | Low | 80 |  |  |  |  |  |  |  |
| Foxp3^+^ |  |  | 0.64 | 0.132 | 0.36 - 1.14 |  |  |  |  |
|  | High | 21 |  |  |  |  |  |  |  |
|  | Low | 69 |  |  |  |  |  |  |  |
|  |  |  |  |  |  |  |  |  |  |
| nTOB1^+^ Foxp3^+^ |  |  | 0.65 | 0.105 | 0.38 - 1.09 |  |  |  |  |
|  | High | 47 |  |  |  |  |  |  |  |
|  | Low | 43 |  |  |  |  |  |  |  |
| cTOB1^+^Foxp3^+^ |  |  | 0.64 | 0.095 | 0.37 - 1.08 |  |  |  |  |
|  | High | 50 |  |  |  |  |  |  |  |
|  | Low | 40 |  |  |  |  |  |  |  |
| CD20^+^ |  |  | 0.57 | 0.042 | 0.33 - 0.98 |  | 0.61 | 0.35 - 1.04 | 0.071 |
|  | High | 51 |  |  |  |  |  |  |  |
|  | Low | 39 |  |  |  |  |  |  |  |
| nTOB1^+^CD20^+^ |  |  | 0.7 | 0.201 | 0.4 - 1.21 |  |  |  |  |
|  | High | 24 |  |  |  |  |  |  |  |
|  | Low | 66 |  |  |  |  |  |  |  |
| cTOB1^+^CD20^+^ |  |  | 0.85 | 0.592 | 0.47 - 1.53 |  |  |  |  |
|  | High | 20 |  |  |  |  |  |  |  |
|  | Low | 70 |  |  |  |  |  |  |  |
| CD68^+^ |  |  | 0.67 | 0.125 | 0.4 - 1.12 |  |  |  |  |
|  | High | 24 |  |  |  |  |  |  |  |
|  | Low | 66 |  |  |  |  |  |  |  |
| nTOB1^+^CD68^+^ |  |  | 0.65 | 0.130 | 0.38 - 1.13 |  |  |  |  |
|  | High | 25 |  |  |  |  |  |  |  |
|  | Low | 65 |  |  |  |  |  |  |  |
|  |  |  |  |  |  |  |  |  |  |
| cTOB1^+^CD68^+^ |  |  | 0.45 | 0.027 | 0.22 - 0.91 |  | 1.53 | 0.34 - 6.93 | 0.582 |
|  | High | 10 |  |  |  |  |  |  |  |
|  | Low | 80 |  |  |  |  |  |  |  |

(Continued)

Supplementary Figures


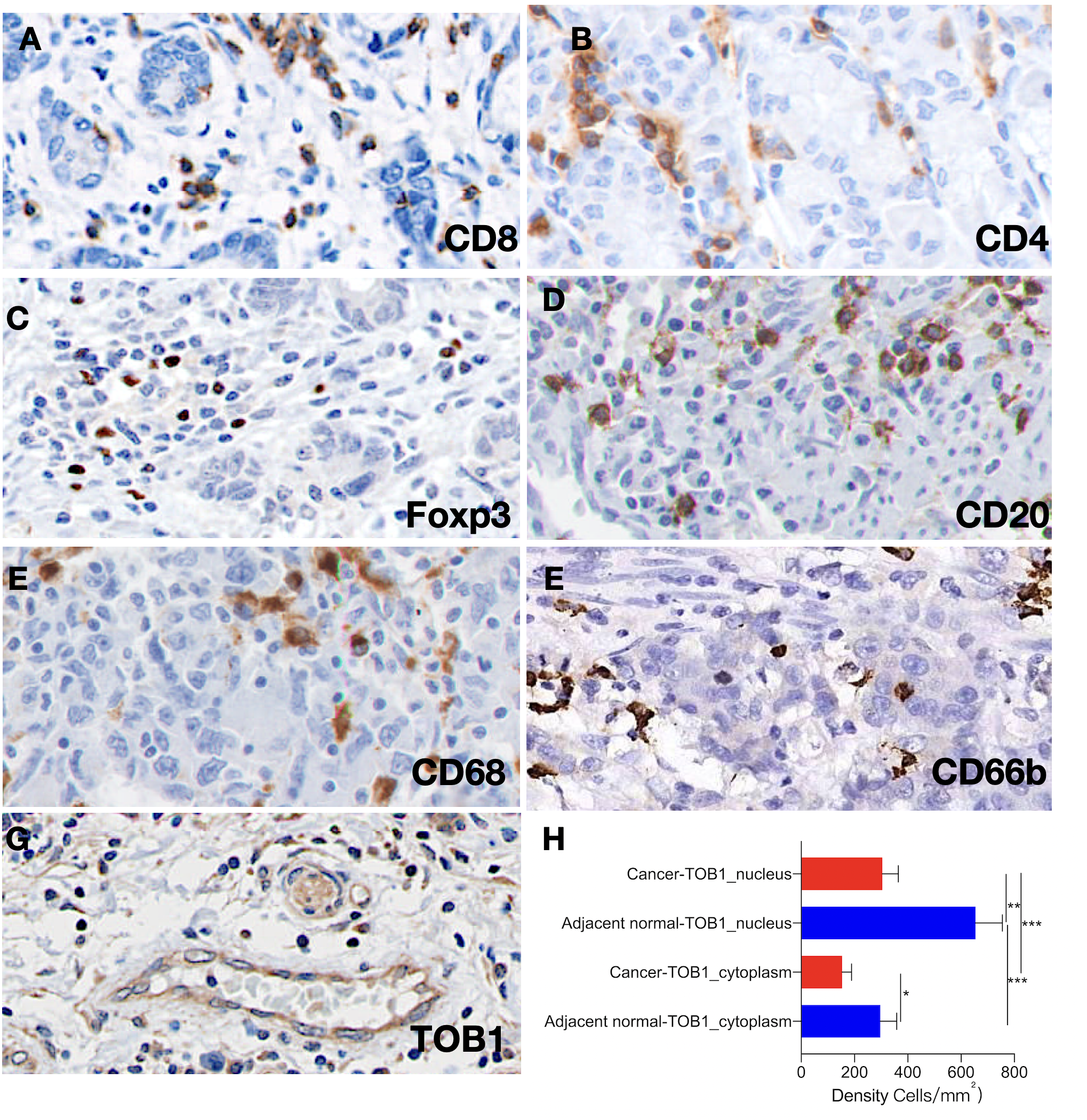


**Figure. S1**: Localization of immune markers and TOB1, as well as the subcellular distribution of TOB1 within tissues. Immunohistochemistry (IHC) was employed to determine the localization of each specific biological marker, CD8(s1A), CD4(s1B), Foxp3(s1C), CD20(s1D), CD68(s1E), CD66b(s1F) and TOB1(s1G). Comparison analysis of cytoplasmic TOB1 and nuclear TOB1 in gastric cancer tissues and adjacent tissues revealed that，regardless of the tissue type, TOB1 expression was predominantly observed in the nucleus (s1H). **P* < 0.05, ***P* < 0.01, ****P* < 0.001.

**Figure. S2**.
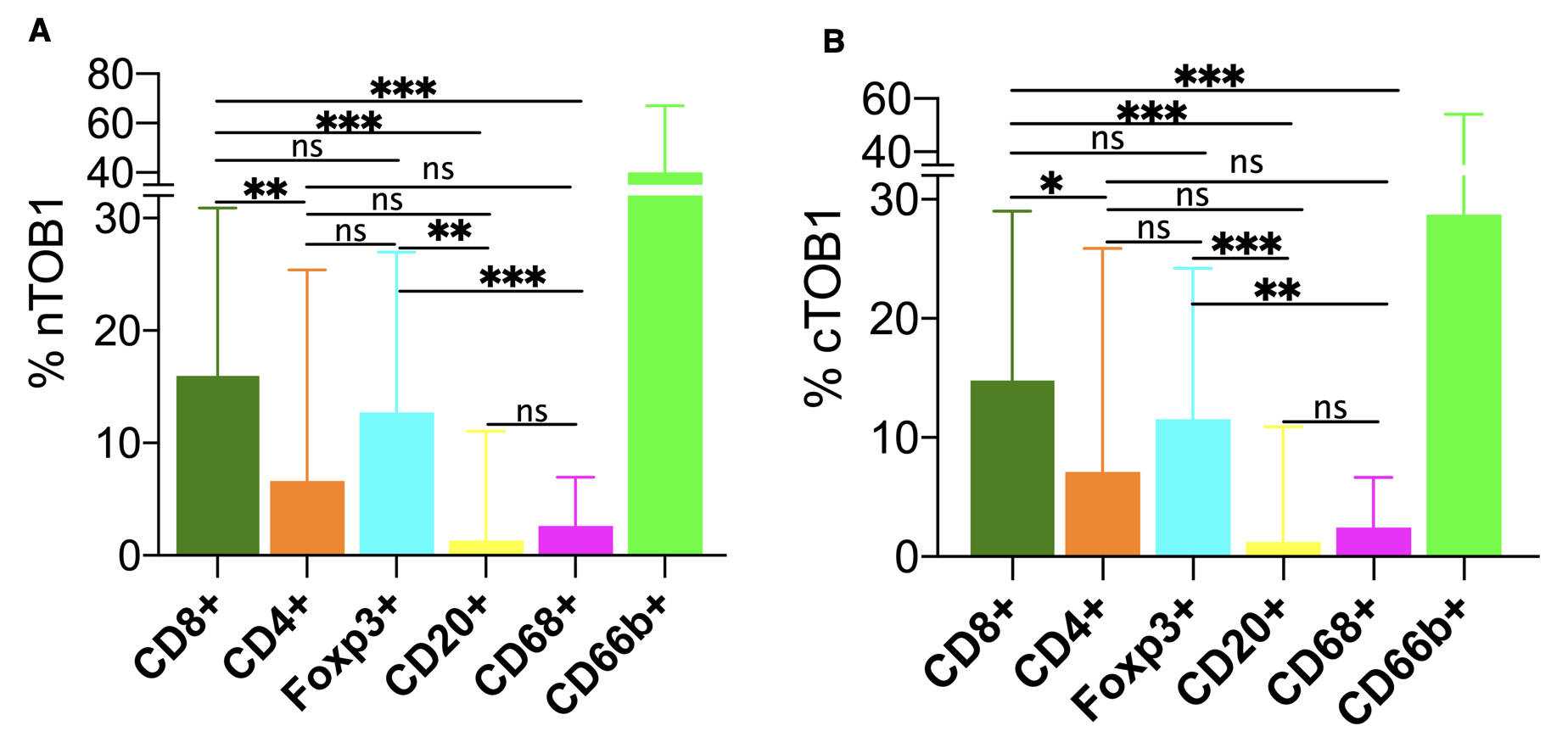
 A-B，the differential expression analysis of nTOB1 and cTOB1 in various immune cells except CD66b^+^ neutrophils were conducted using pairwise comparisons between multiple groups through one-way ANOVA. **P* < 0.05, ***P* < 0.01, ****P* < 0.001, ns, not significant.

**Figure. S3**. Correlation analysis of nTOB1, cTOB1, and the expression levels of six biological markers in gastric Adjacent normal tissues. The lower left section of the figure displays scatter plots depicting the correlations between each pair of indicators. The upper right section provides correlation coefficient (R) values and significance levels. The diagonal section shows the distribution of each individual indicator in bar graphs. **P* < 0.05, ***P* < 0.01, ****P* < 0.001.


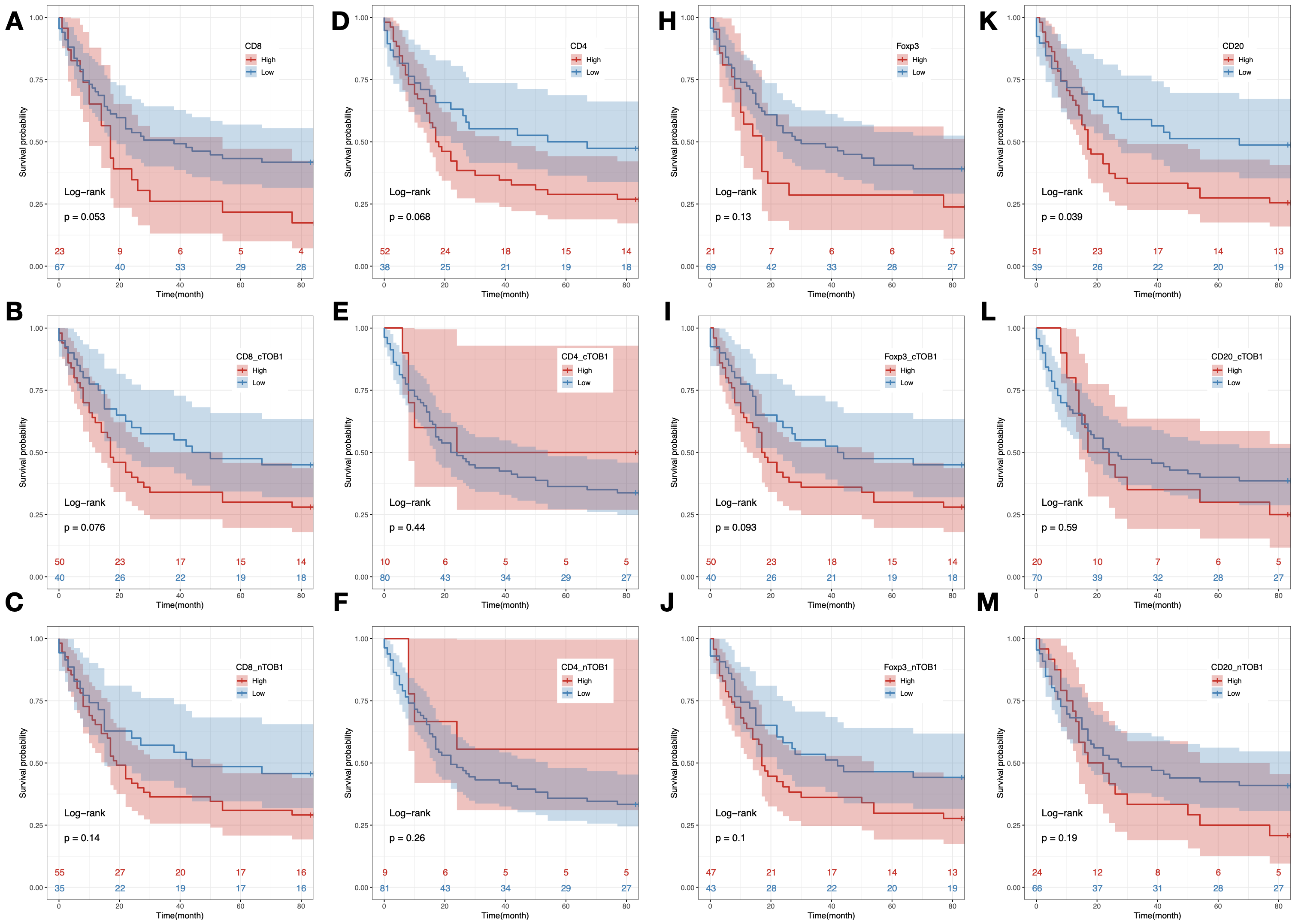


**Figure. S4.** Associations between individual immune cell populations marked with CD8, CD4, FOXP3, CD 20, CD68 or nTOB1/cTOB1 doble positive and patient survival outcomes. Survival probability with high (red line) or low (bule line) infiltration of CD8(S3A_C), CD4(S3D_F), FOXP3(S3H-J) and CD20(D3K-M) in GC specimens.
