# Supplementary material for: TOB1 modulates neutrophil phenotypes to influence gastric cancer progression and immunotherapy efficacy
Source: Front Immunol. 2024 Mar 28;15:1369087. doi: 10.3389/fimmu.2024.1369087 (PMC11010640; doi:10.3389/fimmu.2024.1369087)
Supplement: Supplementary file 2 [file DataSheet_2.docx]

library(limma)

library(ggpubr)

geneFile=" "

immFile=" "

setwd(" ")

rt=read.table(geneFile, header=T, sep="\t", check.names=F, row.names=1)

tumorData=rt[rt$Type=="Tumor",1,drop=F]

tumorData=as.matrix(tumorData)

rownames(tumorData)=gsub("(.*?)\\-(.*?)\\-(.*?)\\-(.*?)\\-.*", "\\1\\-\\2\\-\\3", rownames(tumorData))

data=avereps(tumorData)

immune=read.csv(immFile, header=T, sep=",", check.names=F, row.names=1)

immune=as.matrix(immune)

rownames(immune)=gsub("(.*?)\\-(.*?)\\-(.*?)\\-(.*)","\\1\\-\\2\\-\\3",rownames(immune))

immune=avereps(immune)

sameSample=intersect(row.names(data), row.names(immune))

immune=immune[sameSample,]

immuneTab=cbind(ID=row.names(immune), immune)

write.table(immuneTab, file=" ", sep="\t", quote=F, row.names=F)

library(limma)

library(ggplot2)

library(ggpubr)

cell="Neutrophil_CIBERSORT"

immuneFile=" "

setwd(" ")

rt=read.table(immuneFile, header=T, sep="\t", check.names=F,row.names = 1)

rt=as.matrix(rt)

rt=t(rt)

data=t(rt[cell,,drop=F])

Type=ifelse(data[,cell]>median(data[,cell]), "High", "Low")

data=cbind(as.data.frame(data), Type)

outTab=cbind(ID=row.names(data), data)

write.table(outTab, file=" ", sep="\t", quote=F, row.names=F)

library(limma)

gene=" "

expFile=" "

setwd(" ")

rt=read.table(expFile, header=T, sep="\t", check.names=F)

rt=as.matrix(rt)

rownames(rt)=rt[,1]

exp=rt[,2:ncol(rt)]

dimnames=list(rownames(exp),colnames(exp))

data=matrix(as.numeric(as.matrix(exp)), nrow=nrow(exp), dimnames=dimnames)

data=avereps(data)

data=t(data[gene,,drop=F])

group=sapply(strsplit(rownames(data),"\\-"), "[", 4)

group=sapply(strsplit(group,""), "[", 1)

group=gsub("2", "1", group)

conNum=length(group[group==1])

treatNum=length(group[group==0])

Type=c(rep(1,conNum), rep(2,treatNum))

exp=cbind(data, Type)

exp=as.data.frame(exp)

colnames(exp)=c("gene", "Type")

exp$Type=ifelse(exp$Type==1, "Normal", "Tumor")

exp$gene=log2(exp$gene+1)

outTab=exp

colnames(outTab)=c(gene, "Type")

outTab=cbind(ID=row.names(outTab), outTab)

write.table(outTab, file=" ", sep="\t", quote=F, row.names=F)

library(limma)

library(survival)

library(survminer)

expFile=" "

immuneFile=" "

setwd(" ")

rt=read.table(expFile, header=T, sep="\t", check.names=F, row.names=1)

gene=colnames(rt)[1]

tumorData=rt[rt$Type=="Tumor",1,drop=F]

tumorData=as.matrix(tumorData)

rownames(tumorData)=gsub("(.*?)\\-(.*?)\\-(.*?)\\-(.*?)\\-.*", "\\1\\-\\2\\-\\3", rownames(tumorData))

data=avereps(tumorData)

immune=read.table(immuneFile, header=T, sep="\t", check.names=F, row.names=1)

immuneH=immune[immune$Type=="High",1,drop=F]

immuneL=immune[immune$Type=="Low",1,drop=F]

sameSampleH=intersect(row.names(data), row.names(immuneH))

sameSampleL=intersect(row.names(data), row.names(immuneL))

dataH=data[row.names(immuneH),,drop=F]

dataL=data[row.names(immuneL),,drop=F]

rtH=cbind(immuneH, dataH)

rtL=cbind(immuneL, dataL)

TypeH=ifelse(rtH[,gene]>median(rtH[,gene]), "High", "Low")

rtH=cbind(as.data.frame(rtH), TypeH)

HTab=cbind(ID=row.names(rtH), rtH)

write.table(HTab, file=" .txt", sep="\t", quote=F, row.names=F)

TypeL=ifelse(rtL[,gene]>median(rtL[,gene]), "High", "Low")

rtL=cbind(as.data.frame(rtL), TypeL)

LTab=cbind(ID=row.names(rtL), rtL)

write.table(LTab, file=" ", sep="\t", quote=F, row.names=F)

library(limma)

library(ggplot2)

library(ggpubr)

library(ggExtra)

setwd(" ")

tmb=read.table(" ", header=T, sep="\t", check.names=F, row.names=1)

immune=read.table(" ", header=T, sep="\t", check.names=F, row.names=1)

sameSample=intersect(row.names(tmb), row.names(immune))

tmb=tmb[sameSample,,drop=F]

immune=immune[sameSample,,drop=F]

data=cbind(tmb, immune)

data$TMB[data$TMB>quantile(data$TMB,0.99)]=quantile(data$TMB,0.99)

data$TMB=log2(data$TMB)

data$TypeH=ifelse(data$TypeH=="High", "High-TOB1", "Low-TOB1")

group=levels(factor(data$TypeH))

data$TypeH=factor(data$TypeH, levels=c("Low-TOB1", "High-TOB1"))

comp=combn(group,2)

my_comparisons=list()

for(i in 1:ncol(comp)){my_comparisons[[i]]<-comp[,i]}

boxplot=ggboxplot(data, x="TypeH", y="TMB", color="TypeH",

xlab="High_Neutrophil",

ylab="Tumor Mutation Burden(log2)",

legend.title="",

palette = c("blue", "red"),

add = "jitter")+

stat_compare_means(comparisons = my_comparisons)

pdf(file=" ", width=5, height=4.5)

print(boxplot)

dev.off()

library(limma)

library(ggpubr)

tideFile=" "

riskFile=" "

setwd(" ")

tide=read.table(tideFile, header=T, sep="\t", check.names=F, row.names=1)

group=sapply(strsplit(row.names(tide),"\\-"), "[", 4)

group=sapply(strsplit(group,""), "[", 1)

group=gsub("2", "1", group)

tide=tide[group==0,]

row.names(tide)=gsub("(.*?)\\-(.*?)\\-(.*?)\\-(.*?)\\-.*", "\\1\\-\\2\\-\\3", row.names(tide))

tide=avereps(tide)

risk=read.table(riskFile, header=T, sep="\t", check.names=F, row.names=1)

sameSample=intersect(row.names(tide), row.names(risk))

tide=tide[sameSample, , drop=F]

risk=risk[sameSample, "TypeH", drop=F]

data=cbind(tide, risk)

data$TypeH=ifelse(data$TypeH=="High", "High-TOB1", "Low-TOB1")

group=levels(factor(data$TypeH))

data$TypeH=factor(data$TypeH, levels=c("Low-TOB1", "High-TOB1"))

group=levels(factor(data$TypeH))

comp=combn(group,2)

my_comparisons=list()

for(i in 1:ncol(comp)){my_comparisons[[i]]<-comp[,i]}

for(i in colnames(data)[1:(ncol(data)-1)]){

gg1=ggviolin(data, x="TypeH", y=i, fill = "TypeH",

xlab="High_Neutrophil", ylab=i,

palette=c("#0066FF","#FF0000"),

legend.title="Type",

add = "boxplot", add.params = list(fill="white"))+

stat_compare_means(comparisons = my_comparisons)

pdf(file=paste0(" ", i, ".pdf"), width=5, height=4.5)

print(gg1)

dev.off()

}

library(limma)

library(plyr)

library(ggplot2)

library(ggpubr)

tideFile=" "

immuneFile=" "

setwd(" ")

tide=read.csv(tideFile, header=T, sep=",", check.names=F, row.names=1)

tide$Responder=ifelse(tide$Responder=="True", "Responder", "Non-responder")

group=sapply(strsplit(row.names(tide),"\\-"), "[", 4)

group=sapply(strsplit(group,""), "[", 1)

group=gsub("2", "1", group)

tide=tide[group==0, ,drop=F]

row.names(tide)=gsub("(.*?)\\-(.*?)\\-(.*?)\\-(.*?)\\-.*", "\\1\\-\\2\\-\\3", row.names(tide))

tide=cbind(id=row.names(tide), tide)

immune=read.table(immuneFile, header=T, sep="\t", check.names=F, row.names=1)

immune$TypeH=factor(immune$TypeH, levels=c("Low", "High"))

sameSample=intersect(row.names(tide), row.names(immune))

tide=tide[sameSample, , drop=F]

immune=immune[sameSample, "TOB1", drop=F]

data=cbind(tide, immune)

type=levels(factor(data[,"Responder"]))

comp=combn(type, 2)

my_comparisons=list()

for(i in 1:ncol(comp)){my_comparisons[[i]]<-comp[,i]}

boxplot=ggboxplot(data, x="Responder", y="TOB1", fill="Responder",

xlab="High_Neutrophil",

ylab="TOB1",

legend.title="Responder",

palette=c("blue","red")

)+

stat_compare_means(comparisons=my_comparisons)

pdf(file=" ", width=5, height=4.5)

print(boxplot)

dev.off()
